# Tunneling‐Controlled Fusion of Short‐ and Long‐Term Memory in SiO_2_/HfO_2_‐Based Neuromorphic Device for Time‐Series Prediction

**DOI:** 10.1002/advs.202517994

**Published:** 2025-11-16

**Authors:** Chengdong Yang, Lihua Xu, Linlin Su, Yue Wu

**Affiliations:** ^1^ School of Electronic Information Engineering Wuxi University Wuxi 214105 China

**Keywords:** adaptive switching, neuromorphic devices, STM/LTM fusion, trend prediction, tunneling control

## Abstract

Prediction is a needful action before decision‐making, typically related to the interaction of historical information and current states. It requires computing with information on multiple timescales, meaning intrinsic dynamics in short‐long‐term plasticity. However, current implementations of short‐long‐term memory are still limited by unstable retention in LTM mode and high energy consumption. Here a trap‐assisted tunneling strategy is used in Schottky‐based optical synapse, achieving the tunneling‐switched short‐long‐term memory transformation. Based on the devices, the spike‐timing‐dependent plasticity and spike‐number‐dependent plasticity have been programmed with a low switching energy of 1 pJ that is close to the human brain. By adjustment of light‐pulse inputs, the adaptive short‐to‐long‐term transition can be achieved with a clear mode jump. Furthermore, the formed LTM both in stability (only 0.2 % loss ratio over a span of 50 s) and energy requirement (as low as 3.05 × 10^−11^ W) shows desired improvement compared to other devices implementing short‐long‐term memory. By further mapping device parameters to a long short‐term memory network, it allows for the analysis time‐series data and then to make precise long‐term predictions with an error ratio of as low as 4.465 %. This research can offer a hopeful solution to address the precise trend prediction, holding substantial promise for intelligent applications.

## Graphical abstract

Based on trap‐assisted tunneling, the devices can fuse STM/LTM, where the low switching energy of 1 pJ and stable low‐power retention (0.2 % loss ratio and 3.05 × 10^−11^ W) is achieved. Training in a long short‐term memory network it allows to analysis time‐series data and then makes precise long‐term predictions with an error ratio of 4.465 %.

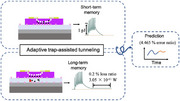

## 1 Introduction

In the human brain, there are two intrinsic memory modes, namely short‐term memory (STM) and long‐term memory (LTM). STM denotes the temporary storage (milliseconds to seconds),^[^ ^1^ ^]^ responsible for computational functions based on current information;^[^ ^2^ ^]^ while LTM lasts for more than an hour, which is key for the encoding of historical information and association.^[^ ^3^ ^]^ The adaptive transition between them is a training‐related dynamics process in which the essence is forming an interconnected loop between specific brain regions that store temporary and lasting memory. This short‐long‐term memory network plays a critical mechanism for high‐tolerant decision‐making via the overall consideration on multiple timescales. Inspired by this, the dual‐mode synaptic devices, with their computing ability over multiple timescales, can help it to analyze time‐series data for making accurate state‐prediction.^[^ ^4^, ^5^ ^]^ In general, the short‐term plasticity (STP) can be computed via volatile STM,^[^ ^6^, ^7^, ^8^, ^9^, ^10^ ^]^ while the absence of intrinsic LTM limits device the computing of long‐term parameters. Such an issue limits the capacity of neuromorphic devices in iterating historical information for providing decision‐making solutions.^[^ ^4^ ^]^

A major challenge facing synaptic devices when compared with biological systems is that there is no specialized mechanism that holds truly lasting memory. Thus, we need to consider adding another LTM mechanism by which LTM can couple with STM operation via an intrinsic switch. In such way, the STM and LTM can be fused in a single device, providing dynamic weight regulation on multiple timescales. In the previous works, the implementation of short‐long‐term memory generally utilizes size‐dependent stability of filaments and charge trapping‐assisted Fe‐FET with two types of memristive dynamics. In the first approach,^[^ ^5^, ^11^, ^12^ ^]^ weak inputs result in the formation of thin, unstable filaments, leading to volatile switching, whereas high‐intensity inputs create thick, stable filaments, resulting in non‐volatile switching. In the second approach,^[^ ^13^, ^14^ ^]^ the long‐term weights are stored in the ferroelectric domain, while the short‐term weights are in situ dynamically operated in a charge‐trapping domain. However, the investigation of short‐long‐term fused techniques is still in the stage of optimization, with several issues, including unstable retention in LTM mode and high energy consumption. The experimental demonstration in devices needs more exploration.

Lateral back‐to‐back Schottky junctions (B‐B SJ) were previously used to achieve the STP by their SiO_2_ temporary trappings.^[^ ^15^, ^16^, ^17^, ^18^, ^19^ ^]^ In this work, we used a trap‐assisted tunneling strategy in B‐B SJ photo‐synapse with tunneling‐switched short‐long‐term memory transformation. At the device level, the heterogeneous oxide of SiO_2_ (3 nm)/high‐k HfO_2_ that serves as a trapping center plays a critical role in fusing different memory modes. In the weak stimuli, memory dynamics is dominated by short‐term mechanisms induced by SiO_2_ trapping alone. With the increase of trap‐induced photogating field, a tiny fraction of electrons tunnel across thin SiO_2_ layer and then in situ accumulate in HfO_2_ trapping. These tunneling electrons can be latched based on the blocking effects of SiO_2_, therefore achieving the adaptive transition between short‐term to long‐term dynamics. Remarkably, the energy for each switching event is calculated to be as low as 1 pJ that is close to the human brain.^[^ ^20^ ^]^ Furthermore, formed LTM both in stability (only 0.2 % loss ratio over a span of 50 s, retention time above 10^4^ s) and energy requirement (as low as 3.05×10^−11^ W) shows desired improvement compared to other devices implementing short‐long‐term memory. Interestingly, using device parameters to map the weight matrix and forget gate in long short‐term memory network (LSTM) can explicitly address time‐series forecasting problem with an error ratio of low to 4.465 %.

## 2 Results and Discussion

### 2.1 Device Structure and Mechanisms for Dual‐Mode Memory Fusion

**Figure 1:**
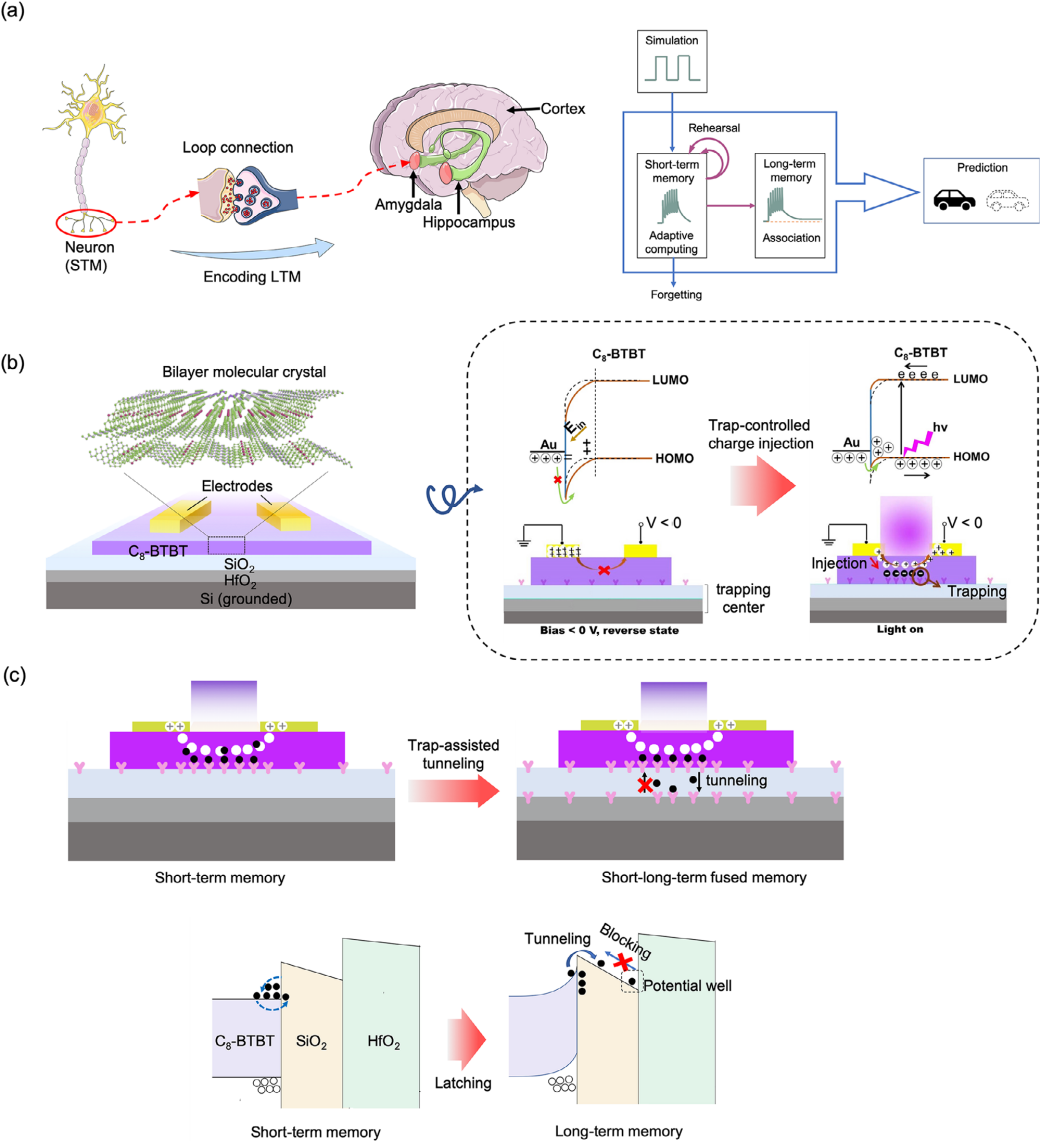
Schematic illustration of the device operating mechanism. a) The schematic illustration of LTM encoding in the human brain. b) The structure and switching mechanism of our device. c) The schematic illustration of memory‐transition mechanism.

Squire proposed that the STM is an intrinsic capacity of each cortical processing system, existing in each brain area.^[^ ^21^ ^]^ Yet there is a substantial difference between the storage regions of short‐ and long‐term memory. The long‐term memory is localized in specific regions of medial temporal (especially hippocampus^[^ ^22^ ^]^ and amygdala^[^ ^21^ ^]^) and diencephalon (see **Figure** **1**a).^[^ ^21^ ^]^ By rehearsal, it can form a complex neural loop between related short‐term neurons and intracerebral regions that is an intrinsic process for encoding and retrieval of long‐lasting memory. LTM is often abstracted as s set of characteristics while maintaining silence if relevant loops are not reactivated in neural activity.^[^ ^3^, ^23^ ^]^ Based on computing with information on multiple timescales, the brain could consciously make predictions and decisions. In analog devices, the B‐B SJ‐based synapses using a heterogeneous trapping layer of SiO_2_/HfO_2_ achieve the coexistence of STM and LTM. The device structure and the mechanism of resistive switching are schematically illustrated in Figure 1b. It should be noted that the 2D (bilayer) molecule‐crystal nature of C_8_‐BTBT is key for the increase of device tunability that underlies the execution of synaptic plasticity. The detailed device fabrication sees Methods.

The device resistance can be optically switched between high‐resistance and low‐resistance states, depending on trap‐controlled dynamics of Schottky barrier (i.e., injection barrier). In dark case, thermodynamic imbalance can shift electrons to the electrode side from HOMO of C_8_‐BTBT, with the forming of charge depletion zone. The C_8_‐BTBT's bands will be downward bent to form a high hole injection barrier (viewed as high‐resistance state). When a 365 nm light is applied, the C_8_‐BTBT can be excited to generate optical charges in which electrons can be easily captured by the trapping layer due to the enhanced interface effect in a nanoscale thickness of C_8_‐BTBT.^[^ ^24^, ^25^ ^]^ The residual holes accumulating at Au side bend the band upward and then reduce the injection barrier (viewed as low‐resistance state, LRS). Importantly to say, the enhancement and transformation of memory are highly relied on the trapping dynamics of charges. As schematically illustrated in Figure 1c, under weak stimuli, the photogenerated electrons are first captured by SiO_2_, featuring short‐term dynamics properties due to the co‐occurrence of charge release. The computing in STP can be implemented in this case, that is, pulse‐programming relaxation dynamics of returning to the initial state spontaneously after removing the stimulus. Furthermore, with the increase of trap‐induced photogating field, the band of C_8_‐BTBT bends upward to build a low enough tunneling barrier for electrons. As a result, a tiny fraction of electrons is able to adaptively tunnel and then reaching at HfO_2_ interface while increasing barrier for electron backflow. Based on this, these electrons can stably be latched at SiO_2_/HfO_2_ interface, in other word, achieving long‐term dynamics properties. Therefore, such trapping/tunneling/blocking structure can theoretically fuse short‐long‐term dynamics via trap‐assisted tunneling.

### 2.2 Film Characteristics and its Electrical Properties

**Figure 2:**
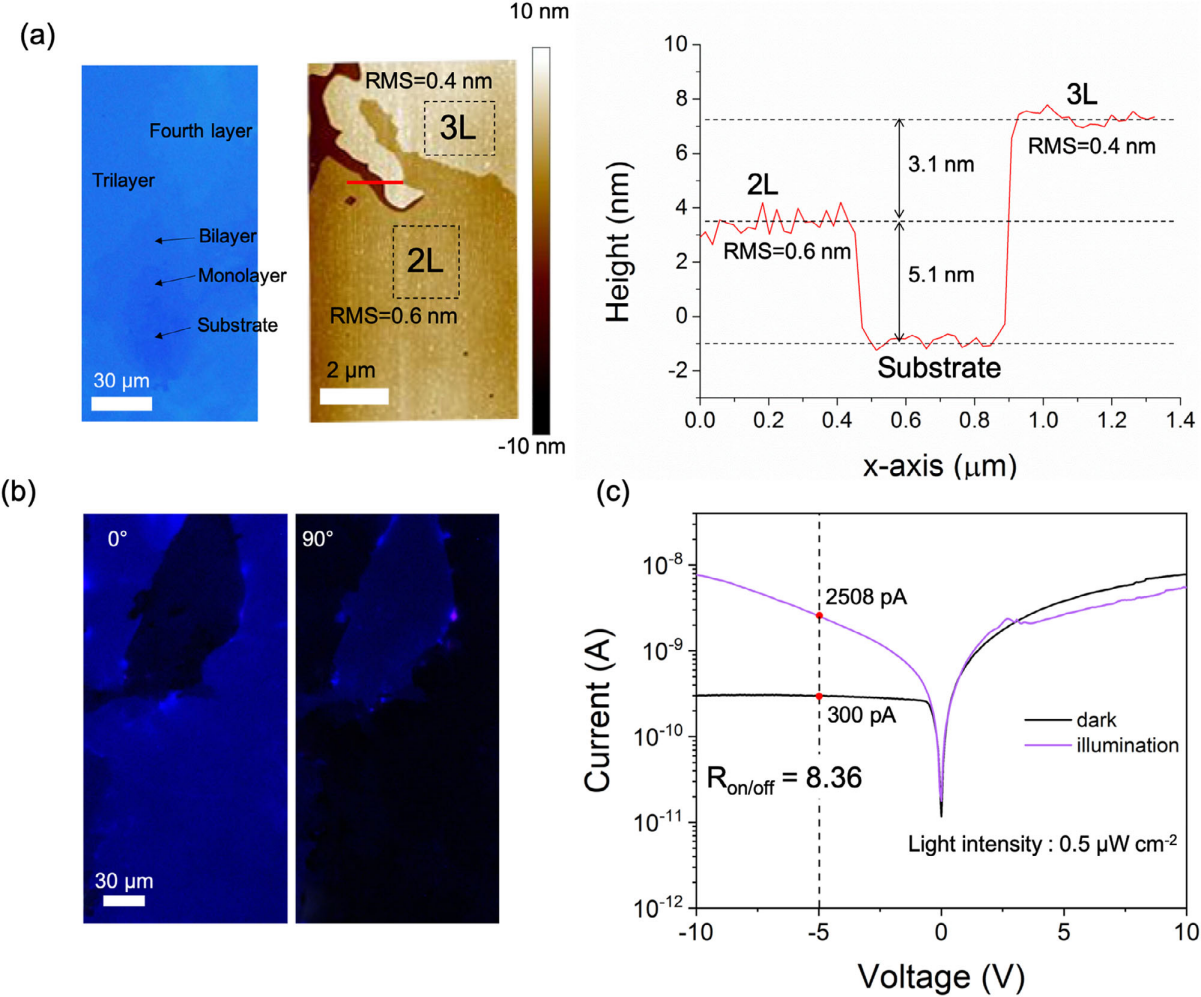
The morphology characterizations and electrical properties of the grown film. a) The optical image (left), AFM picture (middle) of the film, and the step height along the red line (right). b) The cross‐polarized optical images at different sample angles. c) I‐V curve of C_8_‐BTBT/ SiO_2_/HfO_2_‐based B‐B SJ.

A series of film's characterizations have been used to study the 2D nature of C_8_‐BTBT films, a critical property for enhancing trapping effects.^[^ ^19^ ^]^ In **Figure** **2**a, the optical image shows the uniform large‐sized layered film on the micron scale (at left panel). The atomic force microscope (AFM) is used to measure the height of each layer and layer roughness that could illustrate intrinsic alignment (at middle panel). The heights of bilayers (2 L) correspond to the ≈5.1 nm that is consistent with the previous work,^[^ ^26^ ^]^ importantly, it is atomically flat with a roughness of 0.6 nm, indicating an ordered molecular packing structure. In Figure 2b, the cross‐polarized optical images of C_8_‐BTBT films show the uniform brightness change with the sample angle, which is further evidence of ordered molecular alignment. Therefore, our C_8_‐BTBT films demonstrate an atomically smooth 2D features, exhibiting great potential for the achievement of outstanding photoelectric properties.

The *I‐V* scanning was applied to demonstrate the electrical properties in our devices. In Figure 2c, the current shows the unsymmetrical rectifying feature in the dark that is intrinsic in the Schottky contact. Under illumination, the photocurrent rapidly increases in negative bias, while no increase in positive bias, forming the symmetry photocurrent. This transition is the result of transforming Schottky into quasi‐ohmic contact due to the interfacial trap‐controlled charge injection; it is consistent with the switching mechanism mentioned above. Based on this, the photocurrent at ‐1 V (working voltage in synaptic operation) is boosted by ≈8.36‐fold compared with the dark current. The remarkable photocurrent gain is expected to read out high‐quality signals with high energy efficiency.

### 2.3 The STP and the Dynamical Transition Between Memory Modes

**Figure 3:**
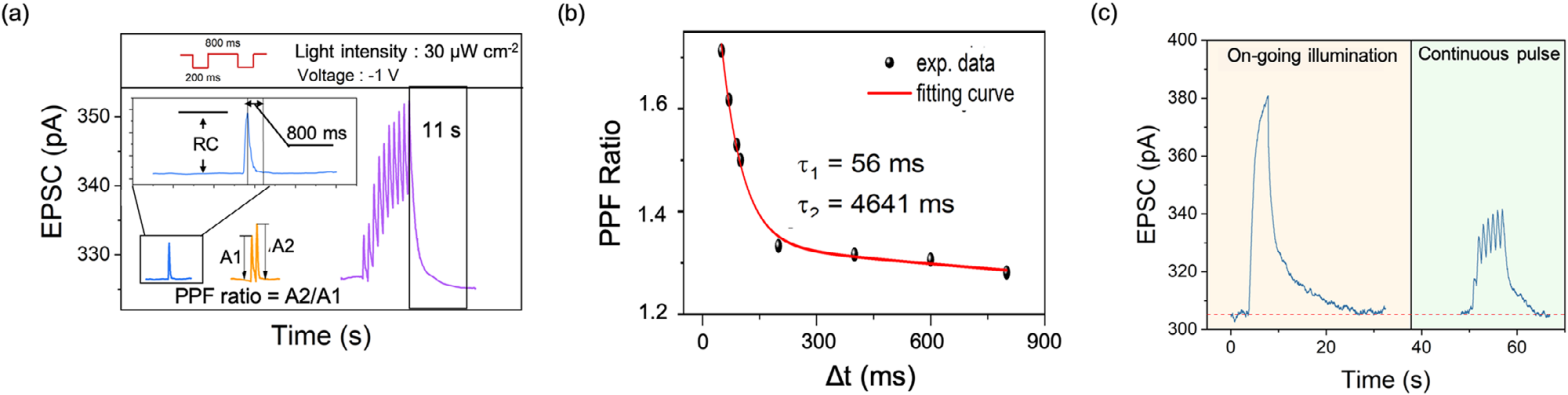
The short‐term plasticity of the device. a) Pulse‐number‐dependent state‐facilitation behaviors. b) The interval‐dependent dynamic PPF behaviors. c) The short‐term features programmed by on‐going illumination and continuous pulses in the device with no HfO_2_.

Next, the detailed discussion of dynamics in plasticity that is characteristic features of synapses will be carried out. As shown in **Figure** **3**a, in single‐pulse test (pulse width is 200 ms, intensity is 30 µW cm^−2^), the spike can be produced with a response current (*RC*) of ≈5 pA, under a low working voltage of ‐1 V. According to it, the switching energy that is the energy dissipated on per switching event, is calculated by

(1) E=V×∫Idt

where *V*, *I* and *t* are the working voltage, response current, and pulse width, respectively. Thus, the energy of our device is measured to be as low as 1 pJ that is more than two orders of magnitude lower than other devices with the short‐long‐term memory (sees **Table** **1**). Notably, the energy density (i. e., energy‐per‐area) is only 2.5 fJ µm^−2^ that is lower than other filamentary or Fe‐FET devices in Table 1. The results compared demonstrate that the charge trapping might be more favorable for energy‐efficient applications.^[^ ^2^ ^]^ The pair‐pulse facilitation (PPF), that the weight is able to be enhanced if a second spike arrives before the vanish of the first spike, could be observed in pair‐pulse test. The PPF ratio is the dynamics in STP dependent on the interval ranging from a few milliseconds to several seconds, and thus regarded as a way to code temporal information.^[^ ^27^ ^]^ In a biological system, the PPF ratio goes the double‐exponential decay with the intervals (*Δt*). The form of decay is

(2) $$PPF\;ratio=1+C_{1}\exp\left(-\Delta t/\tau_{1}\right)+C_{2}\exp\left(-\Delta t/\tau_{2}\right)$$

where the *τ_1_* and *τ_2_* correspond to the characteristic timescales of rapid phase (*F1*) and slow phase (*F2*). In Figure 3b, the PPF ratio of our device can well be fit by a biological form in which both *τ_1_* (56 ms) and *τ_2_* (4641 ms) is close to the biological decay.^[^ ^27^, ^28^ ^]^ The STM can be lengthened by increasing the spike number like that found in biological synapses, where ten‐spike leads to a state‐retention of 11 s while it is only 800 ms after single‐spike, as shown in Figure 3a. In order to evaluate the write endurance that reflects the stability of online learning, we applied 15 circles of write/decay trains where each write course is a pulse sequence containing 60 pulses. As shown in Figure S1 (Supporting Information), during test, the continuous potentiation and decay processes are well repeated, demonstrating good learning stability. Additionally, it should be noted that the control device with no HfO_2_ shows same short‐term dynamics by on‐going illumination or continuous pulses (in Figure 3c); it is evidence for the short‐term dynamics should be originated from temporary charge capture at SiO_2_ trapping. Moreover, as shown in Figure S2 (Supporting Information), both SiO_2_‐only and HfO_2_‐only devices cannot trigger mode transition to produce LTM but only to achieve the dynamics in STP, even though, under high‐intensity stimuli. It indicates that the LTM originates from the heterogeneous dielectric interface of SiO_2_/HfO_2_ that latches tunneling charges.

**Table 1:** Comparison between our work and some state‐of‐the‐art synaptic devices with the short‐long‐term memory.

Materials	Mechanisms	Loss ratio^a)^ or retention time	Switching energy [pJ]	Energy density [fJ µm^−2^]	Power for LTM [W]	Refs.
HZO	Ferroelectric/ Trapping	3.40 %	270	‐	2.13×10^−8^	[4]
Ag_2_S	Metallic Filament	< 400 s	9.92×10^6^	‐	4.96×10^−7^	[11]
Lignin	Carbon‐Rich Filament	9.43 %	2.80×10^5^	35.7	8.4×10^−6^	[12]
MoTe_2_	Ferroelectric/ Trapping	1.43 %	1.20×10^4^	769	8.36×10^−8^	[13]
P(VDF‐TrFE)	Ferroelectric/ Electrochemical	4.33 %	8.97×10^3^	‐	8.84×10^−10^	[14]
WO_X_	Oxygen Vacancy motion	7.69 %	910	5.38×10^7^	2.34×10^−7^	[29]
MoTe_2_/ MoS_2_	Built‐in Field in Heterojunction	5.45 %	8.00×10^4^	1.07×10^6^	2.67×10^−7^	[30]
C_8_‐BTBT	Hybrid Trapping	0.2 %	1	2.5	3.05×10^−11^	This work

^a)^ loss ratio $=\frac{I_{start}-I_{end}}{I_{start}}$, where *I_start_* and *I_end_* are corresponding to start and end signal in a relaxation course.

**Figure 4:**
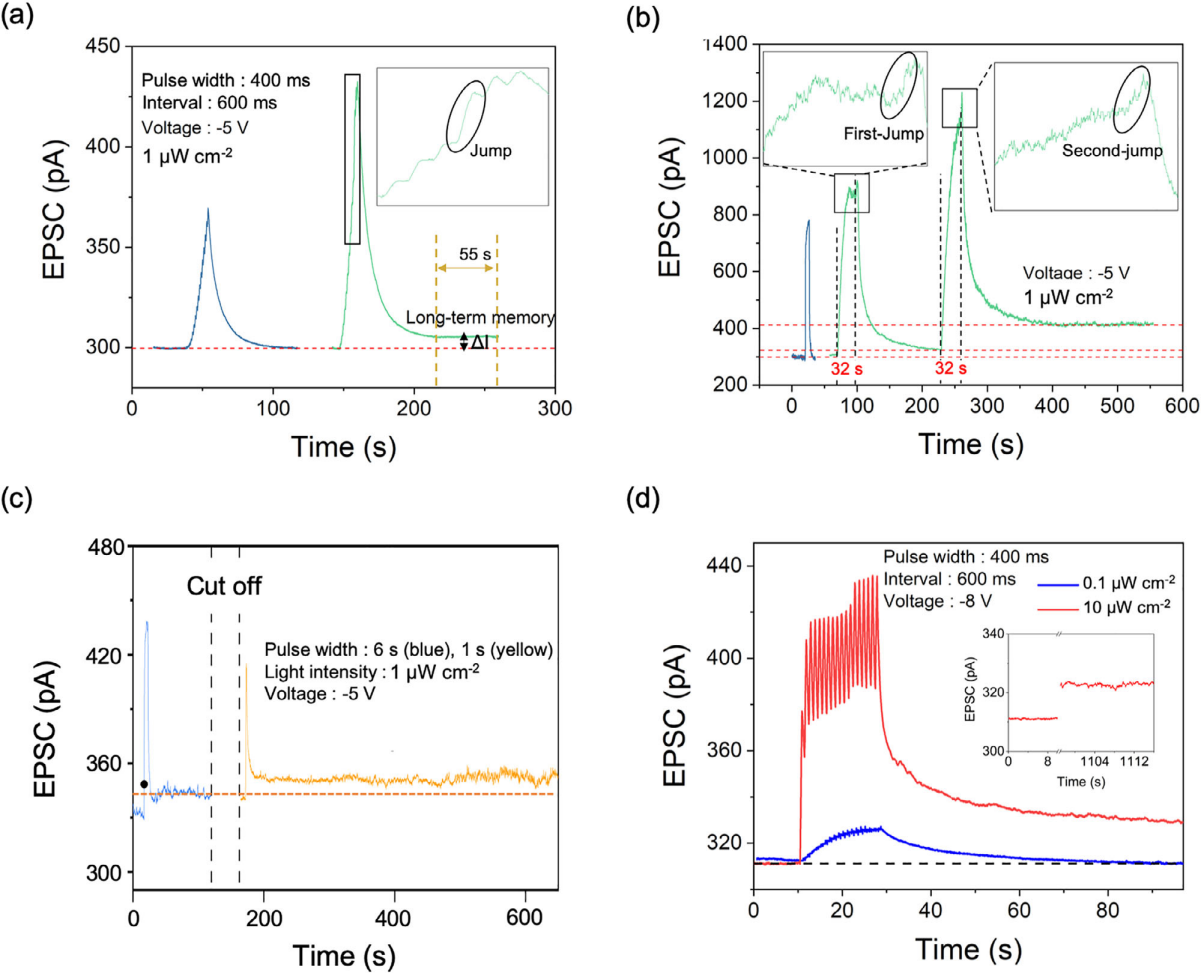
The transitioning of memory modes via different pulse patterns. a,b) The mode change programmed by continuous‐pulse pattern (a) and by lasting‐pulse pattern (b) with the inset showing an enlarged view of the spike. c) The cut‐off test of LTM. The black point is a 2‐s‐width mark. d) The mode transition programmed by an increase of pulse intensity. Inset: the comparison between the initial current and EPSC after 1100 s.

It will be interesting to explore the memory transition between STM to LTM. During continuous‐pulse tests, when the pulse number is not sufficient to form LTM, relaxation dynamics is dominated by SiO_2_ trapping with short‐term attribute (blue line in **Figure** **4**a). If it is enough, the facilitated mode will present a jump (inset in Figure 4a) that accompanies long‐term relaxation (green line in Figure 4a). Such jump is an external expression of tunneling‐switched trapping mode that originates from the accumulation of the photogating field in response to continuous stimuli. In the relaxation course, memory first goes fast decline corresponding to short‐term dynamics of SiO_2_ trapping, and at last holds at a low state‐level rather than continuous loss to initial state, that is a result of latching of a few electrons at SiO_2_/HfO_2_. Such a low state‐level is akin to the LTM keeping silent but not lost, despite in nonactivated neural ensembles. During the 50‐s‐relaxation LTM, the weight shows a loss of only 0.2 % that is a lower level compared to those of other devices with the short‐long‐term memory (sees Table 1). The state‐retention time is above 10^4^ s (sees Figure S3, Supporting Information) where the state after 3 h is still above initial current 10 pA, indicating the robustness and stability of the induced LTM. Such stable state‐retention is of the same order of magnitude compared to that of a previously reported ferroelectric phototransistor.^[^ ^31^ ^]^ Thus, this resulting LTM is stable enough to persist for a long time period, and the stability in conductance is explainable by the blocking effect of SiO_2_. Furthermore, the power for holding LTM was measured in our device, using a formula of

(3) P=ΔI×U

where we define *ΔI* as a current gain compared to initial current, *U* is working voltage. For LTM relaxation with a *ΔI* of ～ 6 pA, the power dissipated is calculated to be 3.05×10^−11^ W, a level smaller than that of other devices in Table 1.

Such fusion of memory dynamics on multiple timescales can be also achieved by wide‐pulse. As shown in Figure 4b, both abrupt jump (see inset) and long‐term relaxation also arise in the use of 32‐s‐pulse, where the resulting state after removing pulses is maintained at a low level of 27 pA above baseline. Interestingly, when the same pulse is applied, the second‐jump can occur with a higher degree that leads to a higher level of retention‐state (110 pA above baseline). It could be explained by which the intrinsic tunneling undergoes the second‐jump, with a higher electron flux than the one presented after first jump. Such multilevel tunneling can provide a solution for more adaptively manage long‐term memory according to external inputs. For comparison, when using a shorter pulse‐width that is not enough for jump, the memory is only short‐term dynamics, verifying that LTM is formed following mode jump. In addition, the formed LTM still exists even when the circuit is cut off, as shown in Figure 4c, where the current level is same before and after it. Hence, the LTM can at once be awakened to in situ accelerate the current weight whenever the pulse is applied again; furthermore, the LTM is also observed to be enhanced by a repeated stimulus, resembling the consolidation during a rehearsal process found in the biological nervous system. The pulse amplitudes are also used to produce the same memory transitioning, as shown in Figure 4d. 18‐pulses with a low amplitude of 0.1 µW cm^−2^ can program dynamics in STP, while the higher amplitude of 10 µW cm^−2^ accelerates enhancement to reproduce both jump and LTM following it. In addition, we found that the formed LTM is reconfigurable, as shown in Figure S4 (Supporting Information), where an almost same LTM formed process (with a jumping characteristic) again develops after first LTM process. It indicates that the transition between STM and LTM is stable and reconfigurable. Figure S5 (Supporting Information) presents PPF ratio, normalized threshold (*I_threshold_*/*I_dark_*), switching energy and LTM level characteristics of 8 individual devices, indicating device‐to‐device variation with coefficient of variation (standard deviation σ‐to‐mean ratio) of 0.05, 0.43, 0.51, 0.54, respectively. This variability may be induced by personal error in device fabrication, as well as by nonuniform SiO_2_ deposited layer, etc. These results demonstrate that, adjusting patterns of light‐pulse input, the short‐long‐term memory can be adaptively fused in single device.

**Figure 5:**
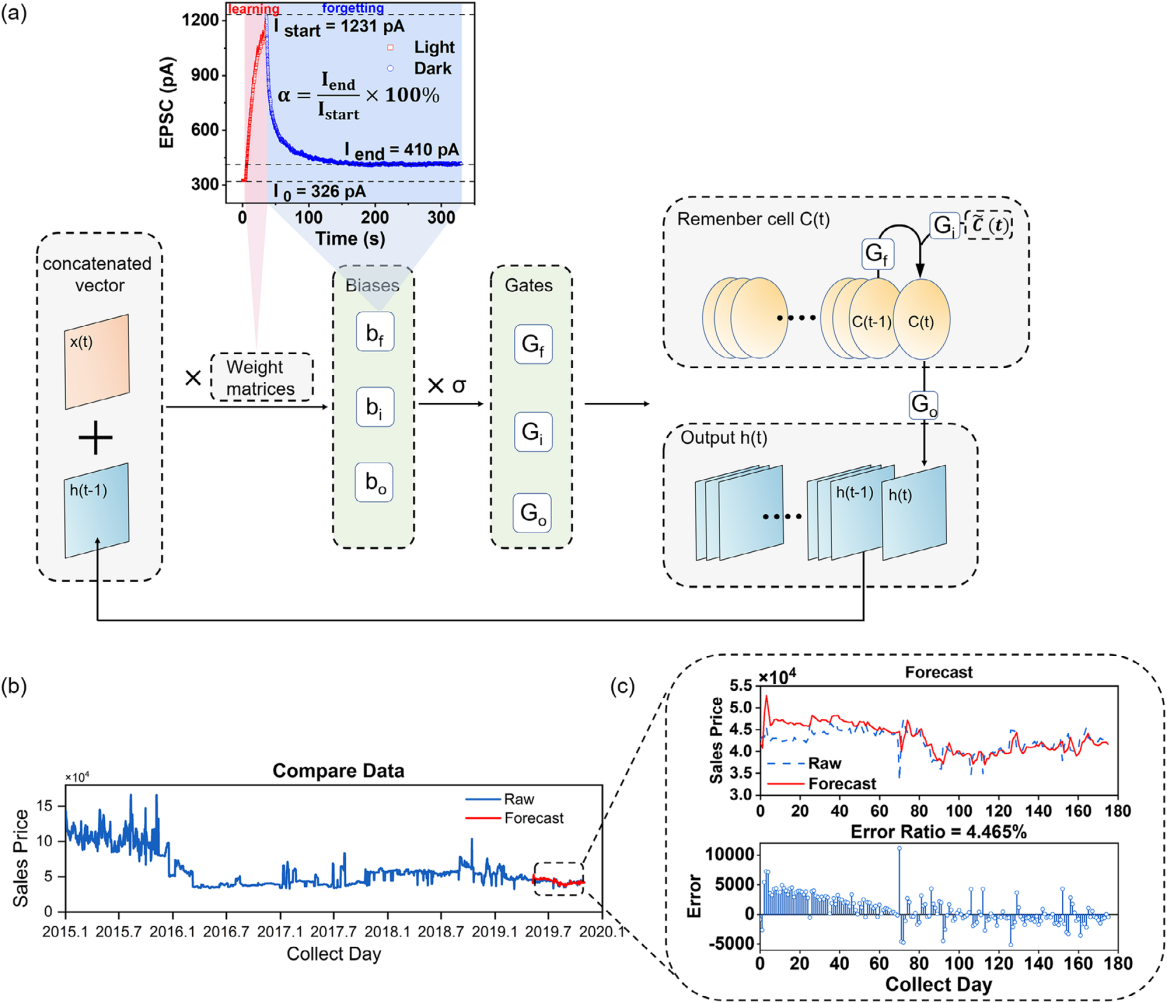
The application of our device in LSTM network for time‐series prediction. a) The diagram of a standard LSTM cell with mapping of our device parameters. b) The forecasting result of model. c) The enlarged drawing of the testing part and its error curve.

As a recurrent neural network (RNN) with feedback connections, LSTM^[^ ^32^, ^33^, ^34^ ^]^ further introduces specialized cells for the memory circulation and uses a gated structure to control the flow of data, allowing it to identify long‐term trends. The diagram of a standard LSTM cell is shown in **Figure** **5**a. It is the algorithmic core idea to use three gates (including input gate *i*(*t*), forget gate *f*(*t*), and output gate *o*(*t*)), in which the *f*(*t*) is a key gate to control the feedback of the previous cell's data. Hadamard multiplication of *f*(*t*) with the previous cell state *C*(*t−1*) is used to decide whether to keep or partially/completely delete information on *C*(*t−1*) in the current cell. These gate values are calculated by the same method that, the concatenated vector, that is comprised of input *x*(*t*) and data from a previous cell *h*(*t‐1*) is multiplied by a weight matrix then added a corresponding bias, and the obtained outputs go through an activation function of sigmoid (*σ*) to form gate values. Therefore, both the weight matrix and bias are thought to be deciding factors for gate values. Herein, the weight updates during device learning are mapped to a matrix. For the value of *b_f_* we extract in the forgetting course using below,

(4) $$\alpha =\frac{I_{end}-I_{0}}{I_{start}-I_{0}}\times 100\%$$

(5) $$b_{f}=\ln\left(\alpha /1-\alpha \right)$$

where *α* is the loss rate, *I_end_*, *I_start_* and *I_0_* are retention degree, learning degree, and initial state, respectively. So, *α* is calculated to be 9.28 % and then obtaining a *b_f_* of ‐2.28.

To evaluate the forecasting performance of the device in LSTM network, we further let the input layer receive a time‐series data of the purchase price of jeans from Korea e‐government website, which contains raw price parameters from 2015.1 to 2020.1,^[^ ^35^ ^]^ as shown in Figure 5b. The network undergoes a nonlinear mapping consisting of 200 hidden units to output the forecasting result. During training, the maximum number of iterations is set to 500, and the initial learning rate is set to 0.02. In the test process, let the training set occupy 90 % and the remaining 10 % is used to assess its forecasting performance. As shown in Figure 5c, model demonstrates high consistency between raw data and forecasting result, with an average prediction error of low to 4.465 %. The model's prediction accuracy is also tested with no use of our device parameters. As shown in Figure S6 (Supporting Information), it is observed that the model cannot precisely forecast the fluctuation, and the error sharply increases to 16.656 %. Therefore, the accuracy improves significantly as using our device, indicating that our device could offer a potential pathway for developing advanced intelligent computing and prediction technologies.

## 3 Conclusion

We have reported a new type of lateral Schottky diode with hybrid oxides that provides two trapping modes for short‐long‐term memory, enabled by trap‐assisted tunneling. First, based on dynamics in STP, several important short‐term features have been optically programmed, including paired‐pulse facilitation, spike‐timing‐dependent plasticity, spike‐number‐dependent plasticity. The energy for each switching event is lowered to 1 pJ, which is close to that in human brain. Second, by adjusting patterns of light‐pulse input, the short‐to‐long‐term transition could be achieved with a clear mode‐jump. Furthermore, formed LTM both in stability (only 0.2 % loss ratio over a span of 50 s, retention time above 10^4^ s) and energy requirement (as low as 3.05 × 10^−11^ W) shows desired improvement compared to that of other memristor devices. By further mapping device parameters to LSTM network, it allows to analyze time‐series data and then to make a precise long‐term prediction with an error ratio of low to 4.465 %.

## 4 Experimental Section

### Growth of 2D Molecular Crystals of C_8_‐BTBT

A piece of Si substrate (roughly ≈1 cm^2^) successively grown 100‐nm‐thick layer of HfO_2_ and 3‐nm‐thick layer of SiO_2_ was cleaned by a standard cleaning process. The HfO_2_ is grown by magnetron sputtering and SiO_2_ is grown by chemical vapor deposition. Then, molecular layer‐defined C_8_‐BTBT molecular crystals were easily prepared on this substrate via the floating‐coffee‐ring‐driven assembly.^[^ ^26^ ^]^ The detailed growth procedure involved the following steps. The growing solvent was first allocated by mixing anisole and p‐anisaldehyde (0.5 %, mass fraction). The C_8_‐BTBT was then dissolved in this mixed solvent to obtain a growing solution. Subsequently, grown solution (3 µL) was dropped onto the pre‐cleaned SiO_2_/HfO_2_/Si substrate and then a pump was used to vent the air. The resulting high‐quality molecular crystals were grown along the pulling track of the liquid drop.

### Device Fabrication

The 2D C_8_‐BTBT film was prepared on a SiO_2_/HfO_2_/Si substrate; subsequently, two Au electrodes with an area of 50 × 40 µm were transferred onto its surface by “stamping” Au stripes with the channel width and length of ≈40 and 10 µm, respectively. Notably, the two Au electrodes should be attached onto the same crystal domain, ensuring the absence of defects from step lines.

## Conflict of Interest

The authors declare no conflict of interest.

## Supporting information



Supporting Information

## Data Availability

The data that support the findings of this study are available from the corresponding author upon reasonable request.
