# Supplementary material for: Tunneling‐Controlled Fusion of Short‐ and Long‐Term Memory in SiO2/HfO2‐Based Neuromorphic Device for Time‐Series Prediction
Source: Adv Sci (Weinh). 2025 Nov 16;13(7):e17994. doi: 10.1002/advs.202517994 (PMC12866721; doi:10.1002/advs.202517994)
Supplement: Supplementary file 1 — Supporting Information [file ADVS-13-e17994-s001.docx]

Supporting Information

Tunneling-Controlled Fusion of Short- and Long-Term Memory in SiO_2_/HfO_2_-Based Neuromorphic Device for Time-Series Prediction

Chengdong Yang, Lihua Xu, Linlin Su,^*^ and Yue Wu


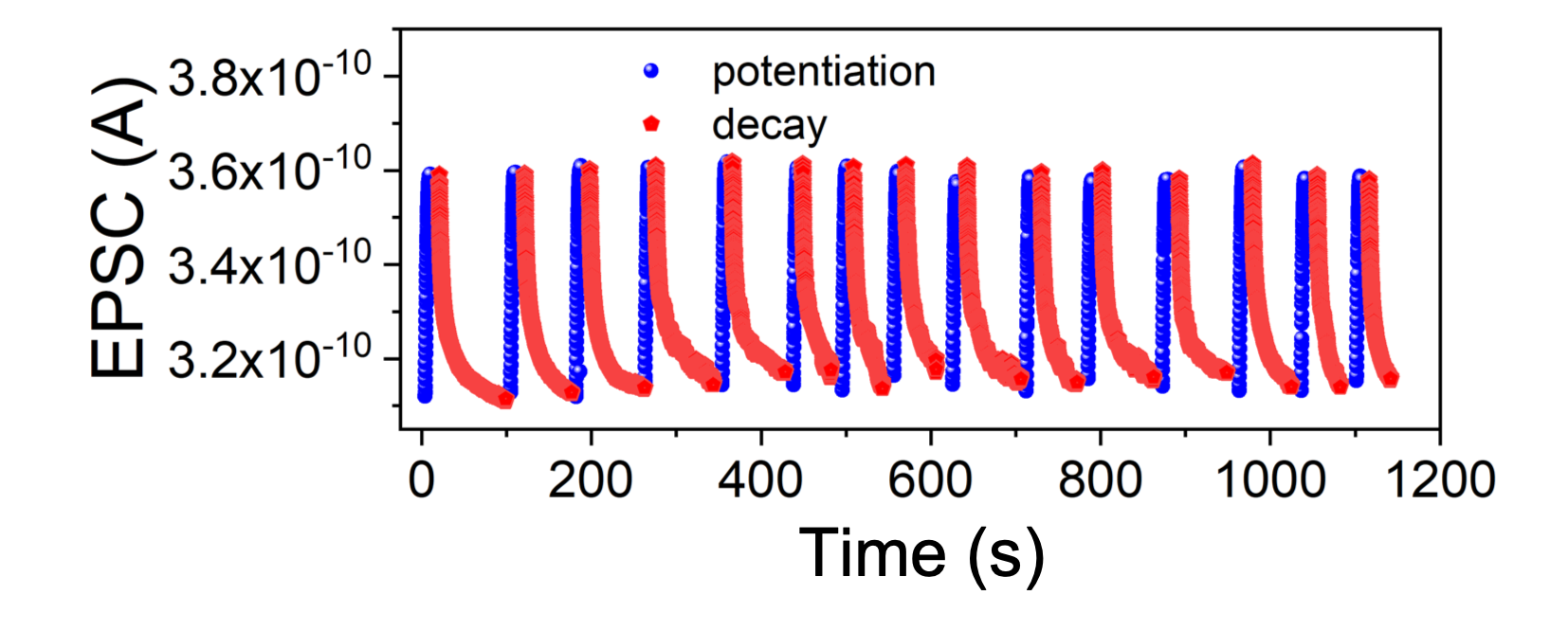


**Figure S1.** The write/decay cycle test under STP mode.


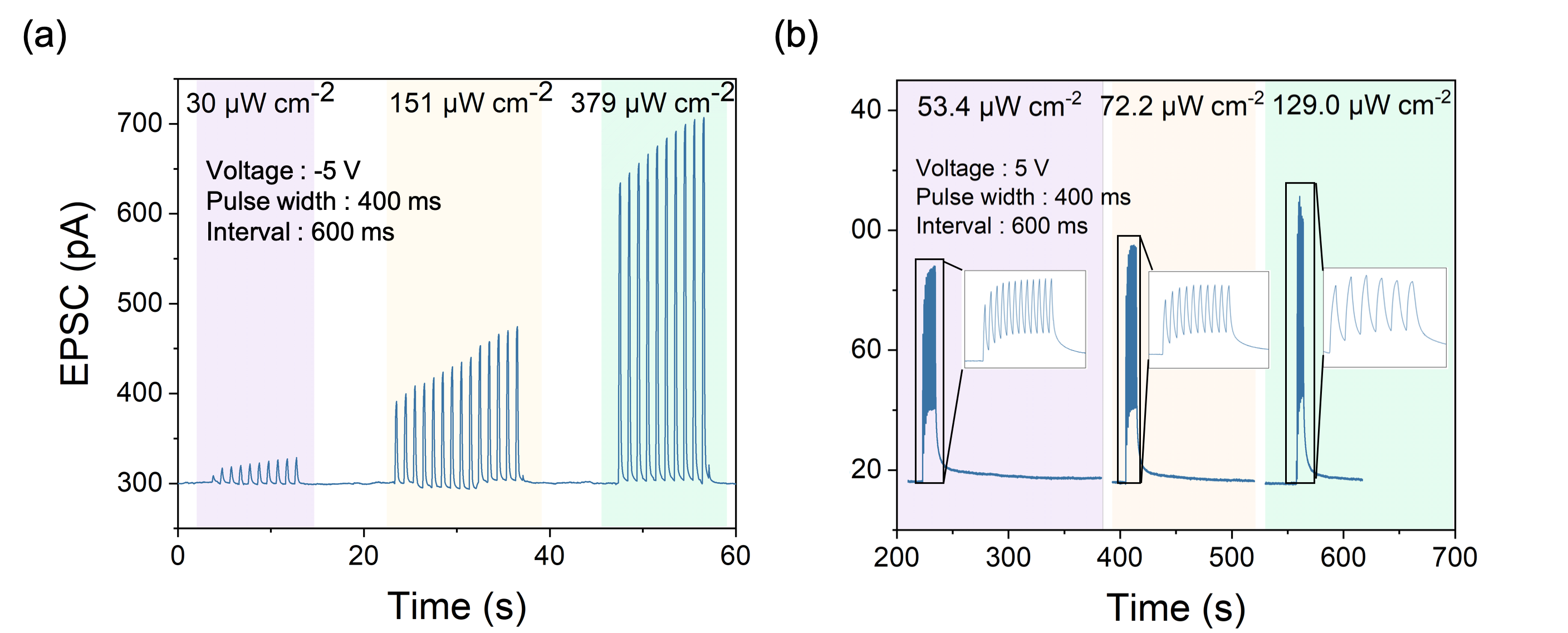


**Figure S2.** The continuous-pulse test under varying light powers for devices utilizing solely SiO_2_ substrates (a) and HfO_2_ substrates (b).


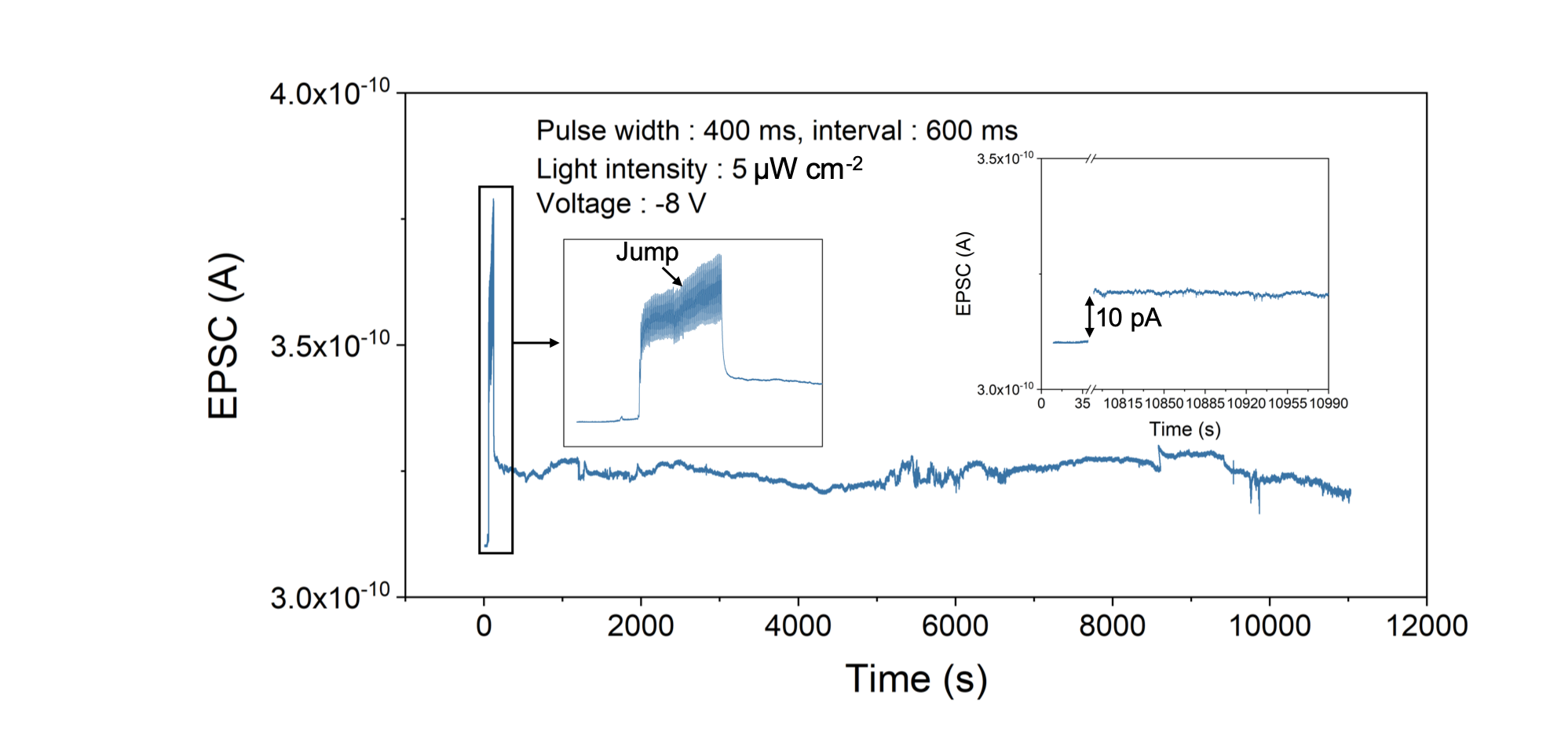


**Figure S3.** The retention time of EPSC. Inset: an enlarged view of the spikes (left) and the comparison between the initial current and EPSC after 3 h (right).


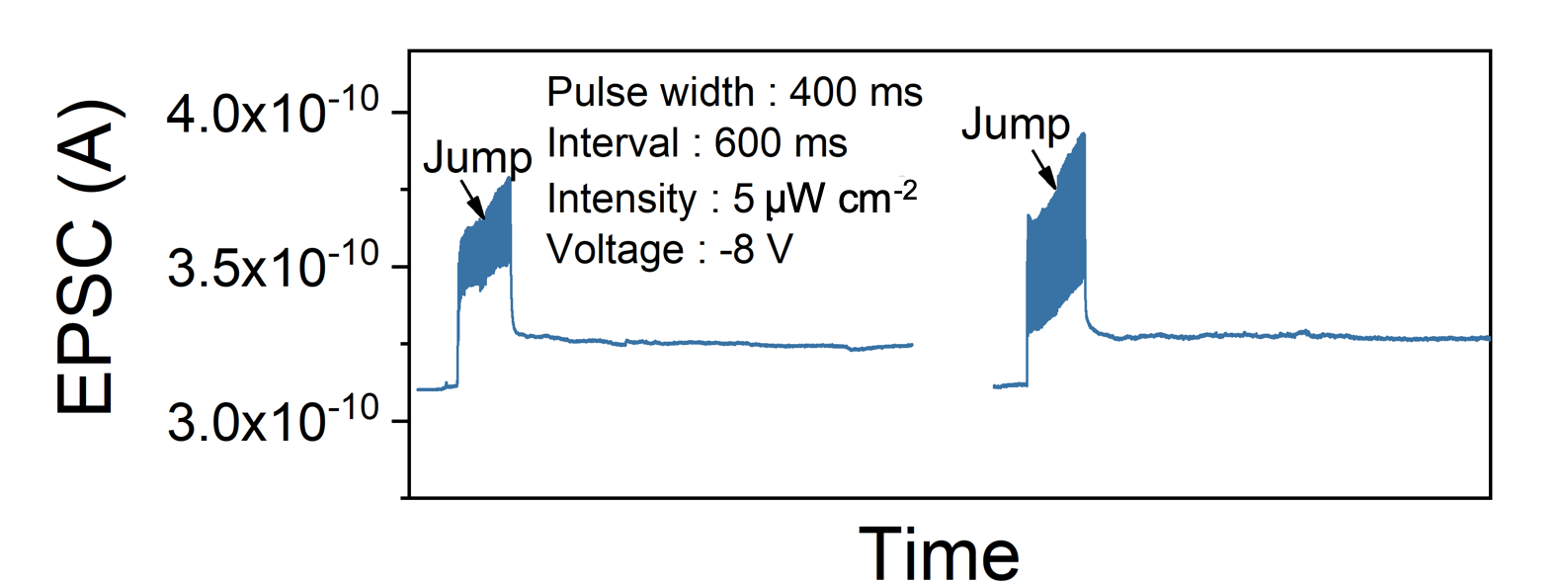


**Figure S4.** The reconfigurability test of the transition from STM to LTM.


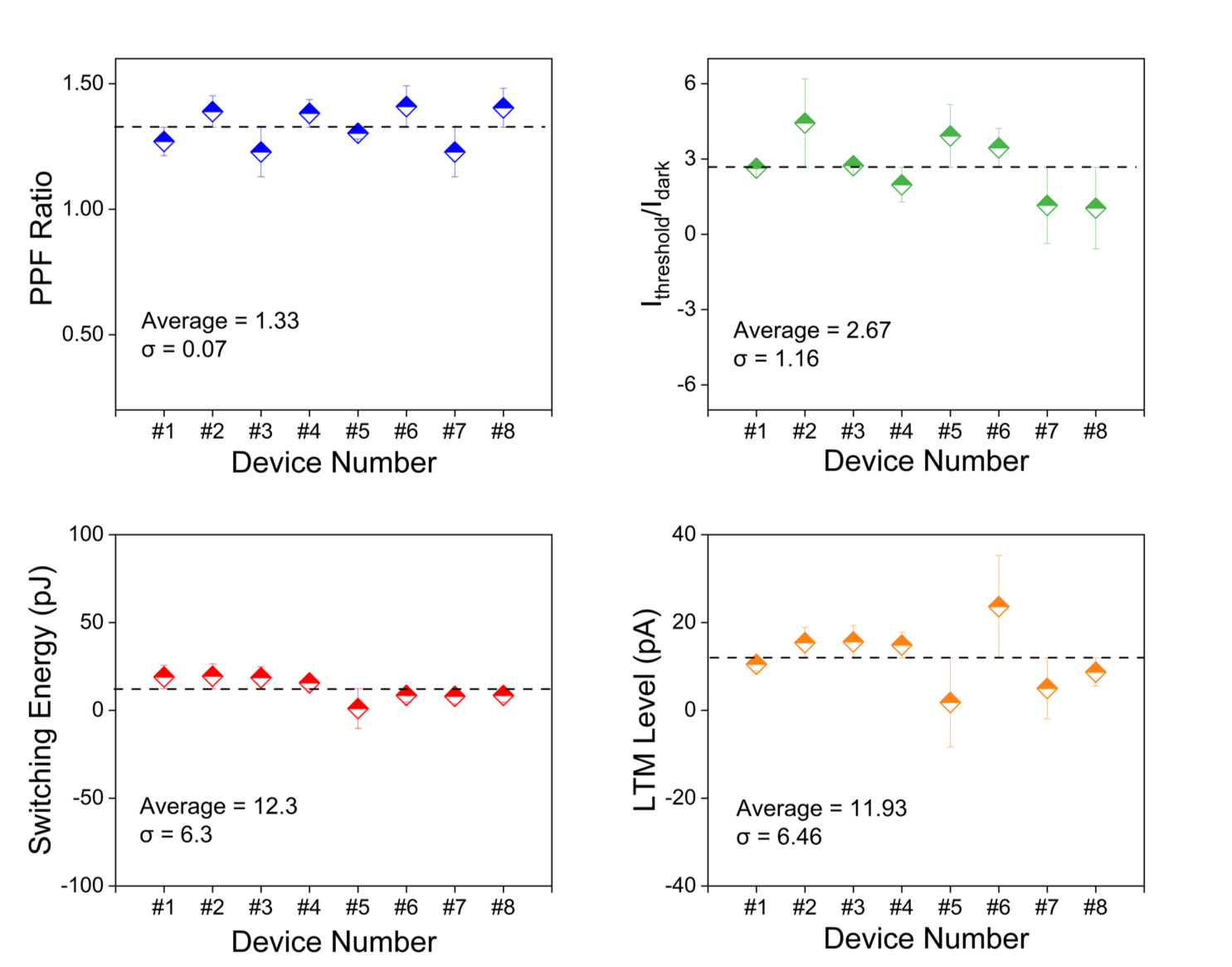


**Figure S5.** The PPF ratio, normalized threshold (*I_threshold_*/*I_dark_*), switching energy and LTM level characteristics from 8 devices.


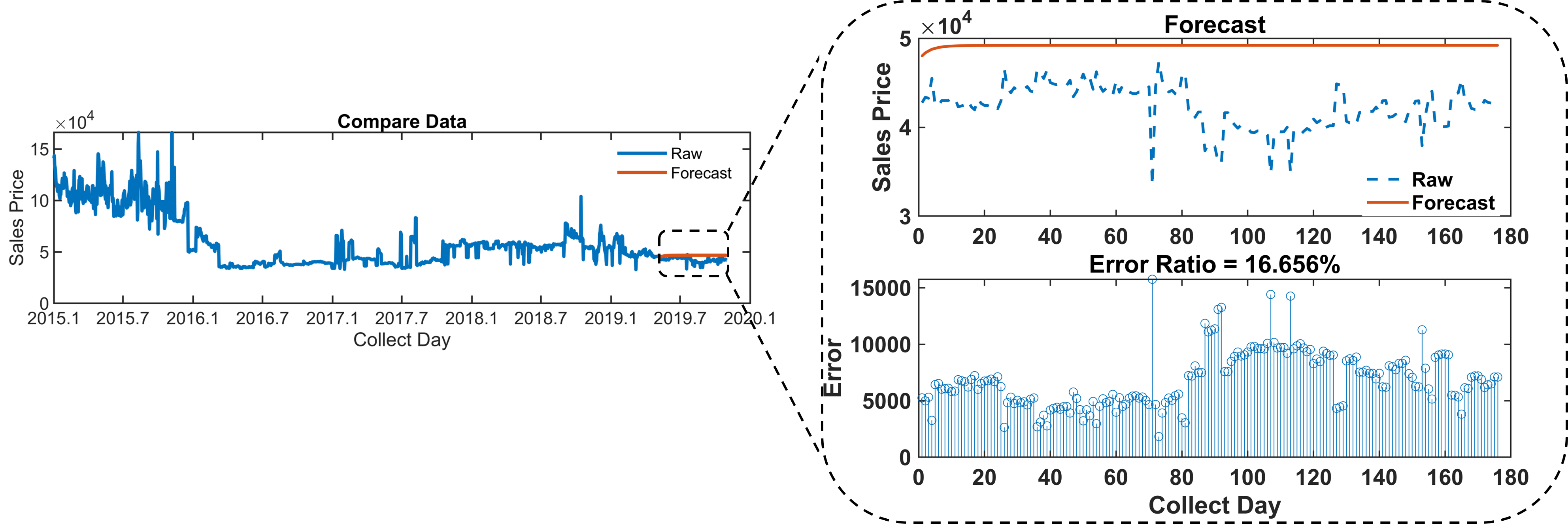


**Figure S6.** The forecasting result of LSTM network unused our device parameters.
